# Myeloid Derived Suppressor Cells: Fuel the Fire

**DOI:** 10.4172/2168-9652.1000e123

**Published:** 2014-06-23

**Authors:** B. R. Achyut, Ali S. Arbab

**Affiliations:** Tumor Angiogenesis Lab, Biochemistry and Molecular Biology Department, Cancer Center, Georgia Regents University, USA

**Keywords:** Myeloid cells, Hypoxia, Suppressor Cells, Bone marrow, Tumor

## Abstract

Low oxygen tension, hypoxia, is a characteristic of many tumors and associated with the poor prognosis. Hypoxia invites bone marrow derived cells (BMDCs) from bone marrow to the site of tumor. These recruited CXCR4+ BMDCs provide favorable environment for the tumor growth by acquiring pro-angiogenic phenotype such as CD45+VEGFR2+ Endothelial Progenitor Cells (EPC), or CD45+Tie2+ myeloid cells. CD11b+CD13+ myeloid population of the BMDCs modulate tumor progression. These myeloid populations retain immunosuppressive characteristics, for example, myeloid derived suppressor cells (MDSCs), and regulates immune- suppression by inhibiting cytotoxic T cell function. In addition, MDSCs were observed at the premetastatic niche of the distant organs in other tumors. Protumorigenic and prometastatic role of the myeloid cells provides a basis for therapeutic targeting of immunosuppression and thus inhibiting tumor development and metastasis.

## Editorial

Bone Marrow Derived Cells (BMDCs) play a pivotal role in tumor microenvironment [1]. Tumor induced changes such as hypoxia is involved in the up-regulation of HIF1-α followed by induction of Stromal Cell Derived Factor-1 Alpha (SDF1α) and secretion of various pro-angiogenic factors and recruitment of CXCR4+BMDCs [2–5]. These recruited cells are characterized as pro-angiogenic CD45+VEGFR2+ Endothelial Progenitor Cells (EPC), or CD45+Tie2+ monocytes [6,7]. Interestingly, lin-ckit+Sca-1+ and their derived cells demonstrate significant recruitment to carcinomas in vivo but they do not functionally contribute to tumor neovascularization [8]. BMPC derived MMP9 modulates neovessel remodeling, thereby playing pivotal role in tumor growth [9,10]. Recent studies have shown that myeloid populations of BMPCs are critical in tumor development [11] e.g. CD11b+CD13+ myeloid cells constitute an immune population of BMPCs that promote angiogenesis, tumor progression and metastasis [12]. Myeloid cells regulate VEGF independent tumor growth and angiogenesis [13]. TGFβ signaling in BMPCs is important and recruits Myeloid Derived Suppressor Cells (MDSCs) via CCL2 in the tumor microenvironment [14]. Additionally, MDSC can be produced in the bone marrow in response to tumor derived factors i.e. Granulocyte Colony Stimulating Factor (G-CSF), IL-6, Granulocyte Monocyte Colony Stimulating Factor (GM-CSF), IL-1β, Prostaglandin E2 (PGE2) and Tumor Necrosis Factor A (TNFα) and are recruited to the tumor site by CXCL12 and CXCL5 [15].

In mice, MDSC express Gr1+ and CD11b+ myeloid markers. Human MDSC express myeloid cell markers such as CD11b+ and CD33+. Monocytic MDSC are usually characterized by HLA-DR−, CD11b+, CD33+ and CD14+ phenotype in humans, whereas mature monocytes express HLA-DR. In mice, monocytic MDSCs express CD11b+Ly6G-/Ly6C+ markers. Granulocytic MDSC are usually characterized by HLA-DR−, CD11b+, CD33+, CD15+ phenotype in humans. Gr1 antigen is absent in the human MDSCs. In mice, granulocytic MDSCs express CD11b+Ly6G+/Ly6Clow markers. Phenotypic characterization of MDSCs is heterogeneous and depends on the site of tumor in human cancers [16]. Signals that stimulate MDSC to acquire immunosuppressive properties are STAT1, STAT3 and STAT6 signal transducer and activator of transcription, and NF-κB transcription factors [17]. Activated MDSC produce Arginase 1 (ARG1), NADPH oxidase, inducible Nitric Oxide Synthase (NOS2), Indoleamine 2,3-Dioxygenase (IDO) and immunosuppressive cytokines that inhibit Cytotoxic T Lymphocytes (CTLs), Dendritic Cells (DC), and Natural Killer (NK) cells [18]. Expression of the B-cell receptor component CD79a on immature myeloid cells contributes to their tumor promoting effects [19]. Downregulation of CD40 expression may contribute to MDSC accumulation by facilitating MDSC resistance to apoptosis [20]. In addition, MDSCs secreted factors expand CD4+CD25+FoxP3+ regulatory T cells (Tregs) to generate immunologically suppressive tumor microenvironment [21].

MDSCs acquire Endothelial Cell (EC) properties in tumor microenvironment and promote tumor growth [22]. MDSC may impair the efficacy of cancer vaccines [23] and antiangiogenic therapy [24]. Peripheral blood MDSC levels associate with a higher tumor burden and a worse prognosis [25,26]. In addition, Gr-1+CD11b+ MDSCs are significantly increased in lungs of mice bearing mammary adenocarcinomas before tumor cell arrival. In the premetastatic lungs, these immature myeloid cells significantly decrease IFN-gamma production and increase proinflammatory cytokines [27]. TGF-β signaling in myeloid cells is required for tumor progression [28] and metastasis [29]. In mice, stromal abrogation of TGF-beta signaling induced accumulation of MDSCs in fore stomach tumors [30]. Thus, protumorigenic and prometastatic role of the myeloid cells provides a basis for therapeutic targeting of immunosuppression and thus inhibiting tumor development and metastasis [31]. In patients, uniform methodology such as computational algorithm-driven analysis is necessary for prospective testing of MDSCs as a biomarker before treatment [32].

So far, many MDSC inhibitors have been developed that are categorized into four groups: (1) deactivation of MDSCs, (2) promotion towards differentiated and mature cells from MDSC, (3) inhibition of development of MDSC, and (4) Depletion of MDSCs. Several of these potential inhibitors have shown the minimal to broad effect on MDSCs [31,33–35]. For example, inhibition of MDSCs enhances anti-tumor immunity by increasing responsiveness to interferon stimulation in murine models [36]. Inhibition of tumor-derived prostaglandin-E2 blocks the induction of MDSCs and recovers NK cell activity [37]. However, better inhibitors are required to increase the efficacy of MDSC inhibition and improve the immunosuppressive effect of MDSCs on tumor microenvironment [38].
